# Lived Experiences and Engagement in an Exercise Program for People with Resistant Major Depression: TRACE-RMD Study

**DOI:** 10.3390/healthcare14070832

**Published:** 2026-03-24

**Authors:** José Etxaniz-Oses, Mikel Tous-Espelosin, Pedro Sánchez, Sara Maldonado-Martín, Ana Isabel Prada-Perea, Nagore Iriarte-Yoller

**Affiliations:** 1 Gizartea, Kirola eta Ariketa Fisikoa Ikerkuntza Taldea (GIKAFIT), Society, Sports, and Physical Exercise Research Group, Department of Physical Education and Sport, Faculty of Education and Sport, Physical Activity and Sport Sciences Section, University of the Basque Country (UPV/EHU), 01007 Vitoria-Gasteiz, Spain; jose.etxaniz@ehu.eus (J.E.-O.); sara.maldonado@ehu.eus (S.M.-M.); 2 Physical Activity, Exercise, and Health Group, Bioaraba Health Research Institute, 01009 Vitoria-Gasteiz, Spain; 3 Hospital of Zamudio, Bizkaia Mental Health Network, Osakidetza Basque Health Service, 48170 Bilbao, Spain; pedromanuel.sanchezgomez@osakidetza.eus; 4 Department of Medicine, Faculty of Health Sciences, University of Deusto, 48007 Bilbao, Spain; nagore.iriarteyoller@osakidetza.eus; 5 Osakidetza Basque Health Service, Araba Mental Health Network, Psychiatric Hospital of Alava, 01006 Vitoria-Gasteiz, Spain; anaisabel.pradaperea@osakidetza.eus; 6 New Therapies in Mental Health Group, Bioaraba Health Research Institute, 01009 Vitoria-Gasteiz, Spain

**Keywords:** depression, physical activity, treatment, qualitative, engagement

## Abstract

**Background:** Resistant major depression (RMD) is characterized by persistent depressive symptoms despite adequate pharmacological treatment, leading to functional impairment and increased physical comorbidity. Lifestyle interventions, particularly physical activity, are promising adjuncts, yet factors influencing engagement remain poorly understood. **Methods:** A purposive sampling approach and thematic analysis informed by a socioecological framework were employed to explore participants’ lived experiences after completing a 12-week supervised combined exercise program. Semi-structured interviews were thematically analyzed. **Results:** Engagement was influenced by three main themes: intrapersonal (symptoms, lifestyle, medication, program expectations), interpersonal (family, peers, healthcare professionals), and environmental (program location, schedule, session design) factors. Motivation was shaped by emotional, physical, and social goals, while barriers included fatigue, anhedonia, and side effects of medication. **Conclusions:** Engagement in exercise interventions for RMD is shaped by the interaction of personal, social, and environmental factors. Understanding lived experiences can inform the design of person-centered, sustainable interventions.

## 1. Introduction

Major depressive disorder has a lifetime prevalence ranging from 5% to 17% in the general population, with approximately 30% of individuals failing to respond adequately to conventional treatments, thus meeting the criteria for resistant major depression (RMD) [1,2]. This condition is debilitating, with persistent depressive symptoms despite multiple pharmacological interventions, resulting in substantial functional impairment and comorbidity. It is typically defined as major depressive disorder with poor or unsatisfactory responses to at least two adequate antidepressant trials from different pharmacological classes [3,4,5,6].

Individuals with RMD not only experience emotional and cognitive impairment but also face higher risks of cardiovascular disease, obesity, and metabolic syndrome [7]. Factors such as genetic predisposition, inadequate pharmacological response, substance use, comorbid medical conditions, and unhealthy lifestyles (sedentary behavior and physical inactivity) exacerbate these risks [8].

Lifestyle interventions, particularly physical activity, improve both physical health and psychological well-being in people with major depression and severe mental illness [9,10]. The World Health Organization (WHO) recommends that people with depression follow the same physical activity guidelines as the general population [11]. Evidence supports the efficacy of exercise in RMD, with supervised aerobic and resistance training combined with treatment as usual showing clinically meaningful reductions in depressive symptoms [12,13]. Mechanisms may involve improvements in mitochondrial function, reduced oxidative stress, enhanced neuroplasticity, and increased cellular energy production [14].

Despite these benefits, dropout rates in exercise interventions for severe mental illness often exceed 25% [15]. There is a critical need to understand the mechanisms underlying participation and adherence, as well as the conditions required for effective program implementation [16]. Qualitative research is particularly suitable for exploring participants’ lived experiences, motivations, and barriers [17].

People with RMD often present with anhedonia, fatigue, cognitive deficits, low self-efficacy, and hopelessness, which impede engagement [2]. Conversely, factors such as mood improvement, stress reduction, social connection, and sense of achievement act as strong motivators [18,19]. Program features, including supervision, individualized progression, and supportive environments, may further enhance adherence [20].

Recent studies also emphasize that lifestyle psychiatry, integrating physical activity, diet, sleep, and social engagement, provides a holistic framework for mental healthcare [21]. Moreover, realist syntheses point out that the effectiveness of physical activity services in inpatient mental health settings depends on the interplay between context, mechanisms, and outcomes [16].

Few qualitative studies have examined the subjective perspectives of individuals with RMD participating in structured exercise programs [22]. While previous qualitative research has explored physical activity in populations with general depression or severe mental illness [18], individuals with RMD represent a particularly complex clinical group characterized by chronic symptomatology, pharmacological resistance, and substantial functional impairment [23]. These features may influence both the motivation to initiate physical activity and the capacity to sustain engagement in structured programs.

Understanding participants’ lived experiences is therefore crucial for developing person-centered and sustainable interventions tailored to this population. Accordingly, the present study explores the lived experiences, motivations, and barriers to engagement in a supervised exercise intervention among individuals with RMD. The analysis is framed within a socioecological perspective encompassing intrapersonal, interpersonal, and environmental influences on exercise participation [24].

## 2. Materials and Methods

### 2.1. Design

A qualitative study using a thematic analysis informed by a socioecological framework was conducted to explore participants’ experiences following a 12-week supervised combined exercise intervention. The study focuses on participants’ lived experiences, and the data were analyzed using thematic analysis following the procedures described by Braun and Clarke. The socioecological model considers multiple levels of influence on health behaviors, including individual, interpersonal, and environmental factors affecting participation in physical activity [25]. Semi-structured interviews were used to allow participants to describe their experiences in their own words while addressing key topics such as motivation, barriers, perceived benefits, and contextual influences [26,27].

The interviews lasted approximately 30 min and followed a flexible interview guide addressing key areas such as motivation, barriers, perceived benefits, and contextual influences. Data collection and analysis were conducted concurrently. Thematic saturation was considered achieved when no new codes or themes emerged across three consecutive interviews during the later stages of data collection, and this was confirmed through discussion among the research team [28].

The qualitative component was conducted as a nested study within the TRACE-RMD intervention project [29]. Participants were recruited from those enrolled in the exercise program and who completed the intervention. Baseline clinical and physiological assessments were included to describe participant characteristics and contextualize the sample, rather than to be analytically integrated into the qualitative findings.

### 2.2. Study Participants

The TRACE-RMD study was conducted between May 2023 and February 2025. A total of 24 participants (mean age 55.9 ± 12.2 years; 14 women and 10 men) diagnosed with RMD were recruited through psychiatrists from the Resistant Depression Unit of the Araba Psychiatric Hospital and the Araba Mental Health Network (Basque Country, Spain). Individuals receiving pharmacological treatment do not achieve remission and show poor or unsatisfactory responses to at least two different classes of antidepressants administered at an adequate dosage and for a sufficient duration [8]. The inclusion and exclusion criteria, as well as the study procedures, followed the published study protocol [29]. The sample size was guided by the principle of data saturation. Participants were recruited until no new themes emerged during analysis.

The study was approved by the Ethics Committee of the local hospital (11 May 2023, Certificate No. 2023–008), and informed consent was explained to, and signed by, all participants before any assessments (ClinicalTrials.gov ID no. NCT05136027). To ensure confidentiality, pseudonyms were used when quoting participants, reflecting their lived experiences and enhancing trustworthiness [30].

### 2.3. Data Collection

The baseline characteristics of the participants, including pseudonym, sex, age, clinical history, and outcome measures—Montgomery-Åsberg Depression Rating Scale (MADRS), Clinical Global Impression (CGI-S), body mass index (BMI), and peak oxygen uptake (VO_2peak_)—are summarized in Table 1. The MADRS and CGI-S were used to assess clinical symptoms in each participant. The MADRS is a clinician-rated scale designed to assess the severity of depressive symptoms across ten items, with scores ranging from 0 (no depression) to 60 (severe depression) [31]. The CGI-S assesses the clinician’s global rating of illness severity on a 7-point scale [32]. Body height and mass were measured to calculate the BMI. Cardiorespiratory fitness (CRF) was evaluated (measured by VO_2peak_) using a symptom-limited cardiopulmonary exercise test on an electronically braked Excalibur Sport cycle ergometer (Lode, Groningen, The Netherlands). R1 and R2 represent individually tailored heart rate zones, with R1 being light-to-moderate (rest to first ventilatory threshold, VT1) and R2 moderate-to-high (VT1 to VT2) intensities. Further details regarding the study protocol have been published [29].

Individual interviews were scheduled according to participants’ availability, in locations chosen collaboratively to foster comfort and equality, and to minimize stigma. A trusting and supportive atmosphere was maintained throughout, emphasizing the value of participants’ contributions. All interviews were conducted face-to-face, audio-recorded with consent, and later transcribed verbatim.

All interviews were conducted by a researcher trained in qualitative methods who was not involved in the delivery of the exercise intervention. This separation helped minimize potential response bias and power dynamics between participants and the intervention team. The interviewer had no prior therapeutic relationship with the participants. Throughout the process, reflexive notes were maintained to document methodological decisions and to acknowledge how the researchers’ professional backgrounds could influence interpretation.

### 2.4. Data Analysis

Data were analyzed using thematic analysis following the procedures outlined by Braun and Clarke [33]. An inductive–deductive approach was adopted. Initial coding was guided by the socioecological framework while remaining open to new concepts emerging from the data [34]. The lead researcher first familiarized herself with the transcripts through repeated readings and developed a preliminary coding framework [34,35].

To enhance analytic robustness, two researchers independently coded the first six transcripts. Coding discrepancies were discussed in depth and resolved through consensus, leading to refinement of the coding framework. The agreed codebook was subsequently applied to the remaining transcripts. Regular cross-checking sessions were conducted to ensure coding consistency and to prevent coder drift. All data management and coding were conducted using NVivo9 (QSR International).

Member checking was conducted with six participants who reviewed a summary of the emerging themes and confirmed that the interpretations reflected their experiences. Reflexive memoing was used throughout the analytic process to document interpretative decisions and enhance transparency.

Codes were subsequently organized into higher-order themes that reflected participants’ perspectives and the aims of the study. Themes were iteratively reviewed, refined, and labeled to capture their underlying meaning and ensure alignment with the socioecological framework.

Methodological rigor was strengthened through the use of multiple strategies. Peer debriefing sessions allowed researchers not involved in data collection to critically examine theme development, thus enhancing credibility. A reflexive stance was maintained throughout the analytic process, with researchers documenting analytic decisions and reflections in memos to acknowledge how their clinical and academic backgrounds could influence interpretation.

These procedures collectively enhanced the dependability, credibility, and confirmability of the findings.

### 2.5. Exercise Program Characteristics

Participants trained twice a week on nonconsecutive days for 12 weeks in a supervised combined exercise program. Each session began and ended with blood pressure monitoring to ensure participant safety. During the sessions, participants were monitored with heart rate monitors (Polar Electro, Kempele, Finland), and the Borg Rating of Perceived Exertion Scale (6–20) was used to optimize and individualize exercise intensity. Each session included a 10 min warm-up and concluded with a 10 min cooldown, both of which featured mobility and stretching exercises. The main part of each session consisted of four 10 min segments: (1) low-volume and low-intensity interval training (LV-LIIT) on a stationary bicycle; (2) a resistance circuit using elastic bands, body weight, and dumbbells; (3) a second LV-LIIT cycling session; and (4) lumbopelvic resistance exercises. All sessions were accompanied by music [29]. Power and speed were modified throughout the sessions to reach the target heart rate. In the aerobic interval training (i.e., LV-LIIT), participants began with a 2 min warm-up at R1 intensity, followed by six intervals consisting of 15 s at R2 and 1 min at R1. The session ended with a 2 min cool-down period at R1. In the resistance circuit training protocol, participants completed 10 strength-resistance exercises, performing each exercise for 30 s followed by 30 s of recovery. The exercises included movements for both the upper and lower body, targeting the main muscle groups, and coordinated with breathing. After finishing the circuit, participants also performed three lumbo-pelvic strengthening exercises (20–25 repetitions per exercise with 30 s of rest between them). Attendance was monitored for each session to assess adherence to the program. The overall mean attendance rate was 85%, with most participants completing the majority of the scheduled sessions.

## 3. Results

Demographic and clinical characteristics of the participants are presented in Table 1.

Three themes emerged from the data analysis: intrapersonal, interpersonal, and environmental factors, and these themes were further divided into several subthemes, which are presented in Table 2 [25], and discussed below.

### 3.1. Intrapersonal Factors

#### 3.1.1. Clinical Characteristics of the Illness

Participants reported long-standing depressive symptoms, often associated with significant life events, occupational stress, or comorbid health conditions. Common experiences included low motivation, persistent sadness, guilt, and reduced autonomy in daily activities. For example, one participant stated: “I don’t feel like doing anything other than lying on the couch… I’m not able to do anything, not even cook” (Lily). Another highlighted feelings of inadequacy and dependence: “I feel guilty… I can’t manage on my own” (James). These chronic symptoms contributed to functional limitations and reinforced physical inactivity, complicating participation in exercise interventions.

#### 3.1.2. Lifestyle

Most participants described largely sedentary routines, including prolonged periods of rest and limited physical activity. Some reported additional risk behaviors, such as smoking: “I still smoke the same. Maybe a bit less” (Abigail). Previous engagement in physical activity varied: Some had participated in sports during childhood or at school, while others abandoned exercise when depressive symptoms worsened: “No, I used to go for walks with some friends. But then I stopped completely, when I started feeling unwell” (Amelia). A few participants maintained regular physical activity, suggesting heterogeneity in baseline lifestyle experiences: “I went hiking a lot… I was always in good physical shape” (Jack).

#### 3.1.3. Medication

Participants’ experiences with pharmacological treatment were mixed. Several reported side effects, including fatigue and reduced motivation: “It makes you lazier… it knocks you down” (Henry); others experienced appetite changes or bloating: “For me, it’s the eating—the anxiety of needing to eat constantly… it has quite a few side effects” (Sophia). A minority reported no noticeable effects: “To be honest, I didn’t notice any side effects” (Oliver). Medication experiences influenced perceived energy and readiness to participate in the exercise program.

#### 3.1.4. Program Perceptions

Initial perceptions of the exercise program ranged from reluctance to enthusiasm. Some participants viewed it as burdensome or obligatory: “At first, I saw it as a burden, having to go twice a week… given the circumstances” (Michael). Others anticipated potential physical or emotional benefits: “Physically, I thought… at least I can strengthen my arms a bit… and my legs too” (Greta); “What I wanted was to get better” (Olivia). Professional recommendations often influenced motivation: “The doctor told me about it, and I said, well, okay” (Amelia). A recurring theme was the use of the program as a self-imposed method to foster action and structure: “Because I was interested, because it was a way to force myself to go and come back” (Samantha). Overall, most participants engaged consistently despite their initial uncertainty or fear.

### 3.2. Interpersonal Factors

#### 3.2.1. External Relationships

Interactions with psychiatrists, family members, and friends played a pivotal role in participants’ engagement. Psychiatrists were often the primary source of referral: “Well, my psychiatrist told me that you were going to run a program like this… and asked if I was interested” (John). Family support contributed to emotional encouragement and accountability: “They encouraged me… ‘Did you go? Did you skip it? Come on, you have to go’“ (Jessica). Conversely, some participants experienced initial isolation or hesitancy in discussing their participation: “I was isolated… My children were living away…” (Henry).

#### 3.2.2. Within-Program Relationships

Participants valued group dynamics, preferring sessions with peers sharing similar conditions, citing empathy, reduced fear of judgment, and mutual support: “I found it interesting to interact with people going through the same thing as me… especially in the early stages” (Jessica). A minority were indifferent to group composition, as the illness itself limited social engagement: “The illness itself greatly restricts social interaction” (George). The role of instructors was emphasized, with participants preferring qualified exercise professionals who could provide guidance and support: “I think it’s the right approach when qualified professionals lead the sessions because you know how to guide us—I wouldn’t be able to do it myself” (David). Instructor gender was irrelevant to participants.

### 3.3. Environmental Factors

#### 3.3.1. Location

Participants expressed divergent preferences regarding program location. Some favored nonhospital environments to avoid negative associations: “Entering that place brings back a lot of memories… I would have preferred a different location” (Sophia). Others preferred the hospital for a sense of security and professional oversight: “I’ve spent a lot of time in the hospital… it made me feel protected” (Amelia). These findings highlight the need for a flexible program design that accommodates individual psychological needs.

#### 3.3.2. Timetable and Duration

Morning sessions were generally preferred due to fatigue in the afternoons: “I think mornings are better—by the afternoon, you’re just kind of ‘blah’” (Sophia). Medication-related morning drowsiness also influenced session timing: “Even now, I still struggle to wake up… sometimes it’s because of the medication” (Samantha). Most participants found two sessions a week over three months manageable, though some suggested longer or more frequent sessions: “Two days a week helped me, but three would’ve been even better… a four-month program would’ve been ideal” (David).

#### 3.3.3. Frequency, Intensity, Time, Type–Progression, Variety (FITT-PV) Principle

The combined aerobic and resistance program was appreciated for its dynamic and varied format. Participants highlighted interval aerobic training and varied resistance exercises as motivating and engaging: “It seemed dynamic to me… your ten minutes of bike and full effort, but then the other thing allowed you a bit more” (Sophia); “The resistance exercises… weren’t the same every day… it’s more entertaining and dynamic” (John). Many perceived the combination of endurance and resistance exercises as a comprehensive and effective approach: “The routine… was fantastic… you gave us everything in the little time we had” (Oliver).

#### 3.3.4. Motivational Elements

Program enjoyment was influenced by session delivery, music, and interpersonal dynamics. Music enhanced engagement and made sessions entertaining: “Yes, I like it a lot” (John); “Yes, it helped… it kept you entertained” (Sophia). Participants reported that structured guidance, supportive peers, and a varied, stimulating environment contributed to continued adherence and intrinsic motivation.

Although the themes were broadly shared across participants, some variability was observed depending on individual characteristics. For example, participants with more severe depressive symptoms at baseline often described greater initial resistance to engaging in the program but also reported experiencing a stronger sense of accomplishment when completing the sessions. Similarly, older participants sometimes emphasized physical health benefits more strongly, whereas younger participants highlighted the psychological and social aspects.

## 4. Discussion

**Figure 1:**
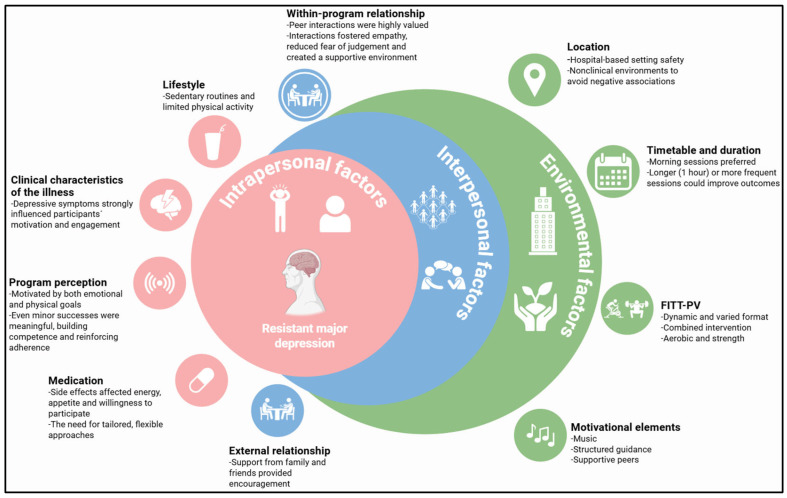
Conceptual representation derived from the thematic analysis illustrating how intrapersonal, interpersonal, and environmental factors interact to influence engagement in the supervised exercise program. Abbreviation: FITT-PV: frequency, intensity, time, type–progression, variety.

This study examined the experiences of individuals with RMD who participated in a supervised exercise program. The findings indicated that engagement is shaped by a complex interplay of intrapersonal, interpersonal, and environmental factors, emphasizing the importance of understanding the lived experiences of this population in order to tailor effective interventions, as shown in Figure 1.

Although the present study did not aim to quantitatively correlate qualitative themes with clinical outcomes, the findings may help contextualize the improvements observed in the broader TRACE-RMD intervention. Participants frequently reported increased motivation, perceived competence, and social support during the program. These factors are consistent with mechanisms proposed in behavioral and motivational theories of exercise adherence, such as Self-Determination Theory, which emphasizes the importance of autonomy, competence, and relatedness in sustaining health behaviors. The qualitative insights, therefore, provide a complementary perspective that may help explain how structured exercise interventions contribute to improvements in depressive symptoms and physical fitness in individuals with RMD.

Intrapersonal factors, particularly the chronic and severe nature of depressive symptoms, strongly influenced participants’ motivation and capacity to engage in exercise [36]. Many described persistent low mood, fatigue, cognitive difficulties, guilt, and a loss of autonomy, which reinforced sedentary behaviors and limited daily physical activity. Previous experiences with physical activity varied: Some participants reported positive past engagement, whereas others had ceased exercise due to worsening symptoms. These results align with research suggesting that RMD is characterized by a high symptom burden, which can impair participation in physical activity [37,38]. Medication side effects further affected energy, appetite, and willingness to participate, illustrating the need for tailored, flexible approaches that account for individual pharmacological experiences [39].

Despite these barriers, participants were motivated by both emotional and physical goals, such as improving their mood, regaining a sense of control, and enhancing physical strength. Even small achievements within the program were described as meaningful, fostering a sense of competence and reinforcing adherence. This result aligns with the growing evidence that physical activity can confer neurobiological benefits in RMD, including increased neurotransmitter synthesis and neuroplasticity, which contribute to mood improvement and functional recovery [21,37,40]. These findings emphasize the value of structuring programs to promote mastery, set achievable goals, and provide positive reinforcement to maintain engagement.

Interpersonal relationships also played a critical role. Psychiatrists served as primary sources of referral, and the trust established within the therapeutic relationship facilitated participation [36]. Support from family and friends provided encouragement and accountability, helping participants overcome their initial reluctance [41]. Within the program, peer interactions were highly valued, particularly in small group settings with participants sharing similar depressive experiences. These interactions fostered empathy, reduced the fear of judgment, and created a supportive environment, highlighting the importance of relatedness in promoting sustained engagement [42,43]. Participants emphasized the role of qualified instructors who adapted sessions to individual needs, thus enhancing feelings of competence and safety, which may contribute to improved adherence [44].

Environmental factors shaped the acceptability of, and adherence to, the program. Location preferences varied, with some participants favoring hospital-based settings for their perceived safety, while others preferred nonclinical environments to avoid negative associations [41]. Morning exercise sessions were generally preferred, reflecting daily energy patterns and medication effects. In this sense, although randomized studies comparing morning to other times are limited and inconclusive, some evidence suggests that physical activity performed in the morning may be associated with improved mood and lower risk of depression, potentially through the alignment of circadian rhythms that act as zeitgebers [45]. Participants also suggested that longer or more frequent sessions could improve outcomes. The dynamic and varied format of combined aerobic and resistance exercises, along with the inclusion of music and choice, enhanced enjoyment and motivation. This issue reflects the importance of providing autonomy-supportive and engaging environments that reinforce mastery and adherence [11,16].

Overall, the results indicate that successful engagement in exercise programs for people with RMD requires a person-centered approach that integrates psychological, social, and environmental considerations. Programs should aim to create positive emotional experiences, foster supportive interpersonal relationships, and provide structured yet flexible exercise sessions that accommodate individual capacities and preferences. These findings are consistent with broader frameworks in lifestyle psychiatry, which advocate incorporating behavioral, psychosocial, and environmental interventions to support recovery and overall well-being in severe mental illness [16,21]. Moreover, evidence suggests that such tailored interventions may be particularly valuable for those for whom conventional therapies often fail to achieve remission [37].

### Limitations and Future Research

Despite providing valuable insights into the experiences of individuals with RMD in a structured exercise program, this study has several limitations. First, data were primarily self-reported, and participants’ reflections may have been influenced by recall bias or social desirability. Second, the relatively wide age range of participants (30–78 years) introduces heterogeneity in physical capacity, clinical history, and social context, which may have influenced individual experiences of the program. Third, participants were recruited through psychiatric referral within a specific healthcare context in the Basque Country, which may limit the transferability of findings to other settings or healthcare systems. Finally, although baseline clinical and physiological measures were collected as part of the broader TRACE-RMD project, the qualitative design does not allow causal inference regarding the effectiveness of the intervention. Additionally, the qualitative sample consisted of participants who completed the intervention, which may introduce a positive participation bias.

Nevertheless, the study also has notable strengths. The use of in-depth interviews provided a nuanced understanding of the complex interplay between intrapersonal, interpersonal, and environmental factors influencing engagement, and maintaining participants’ own words in the analysis enhanced authenticity and preserved the richness of lived experiences.

Additionally, focusing on a population with RMD—a group often underrepresented in physical activity research—addresses an important gap in the literature and offers practical guidance for tailoring interventions to this high-need population. These insights can guide mental health professionals and services in designing more accessible, personalized, and context-sensitive exercise programs. Previous research suggests that exercise in natural environments may enhance motivation and emotional well-being in individuals with mental illness. Future studies could explore whether such contexts influence adherence among individuals with resistant major depression.

## 5. Conclusions

Understanding the lived experiences of individuals with RMD provides valuable insights for designing exercise interventions tailored to this population. The findings suggest that engagement in structured exercise programs is influenced by a complex interaction of intrapersonal, interpersonal, and environmental factors. Programs that incorporate professional supervision, supportive social environments, and flexible exercise structures may facilitate participation among individuals with RMD. Although qualitative in nature, the results highlight the potential role of exercise as a meaningful complementary component within broader treatment approaches for RMD.

## Figures and Tables

**Table 1:** Demographic characteristics of the participants.

Pseudonym	Sex	Age	Onset Age	Hospital Admissions	Occupation Status	MADRS	CGI-S	BMI (kg/m^2^)	VO_2peak_ (mL∙min^−1^∙kg^−1^)
John	M	42	18	0	Incapacity	35	5	26	44
Emily	F	77	35	1	Pensioner	6	2	26.8	8
Amelia	F	54	48	3	Incapacity	40	5	24.9	11
Jack	M	47	37	0	Unemployment	49	6	22.6	25
Lily	F	78	40	2	Pensioner	39	5	31.5	7
James	M	59	27	9	Incapacity	51	6	29.8	15
Sophia	F	35	20	2	Incapacity	11	3	35.2	20
Jessica	F	55	50	0	Incapacity	28	4	26.1	18
Henry	M	62	62	0	Incapacity	23	3	29.2	24
Isabella	F	61	20	0	Incapacity	32	4	39.3	14
Elizabeth	F	30	15	0	Incapacity	32	4	24.9	23
Michael	M	67	66	0	Pensioner	38	5	33.1	18
Margaret	F	57	30	0	Unemployment	44	5	20.3	23
Olivia	F	68	30	4	Pensioner	36	4	21.6	20
Samantha	F	35	33	0	Housewife	36	4	25.7	16
Oliver	M	53	49	0	Incapacity	24	4	35.4	18
George	M	46	41	1	Incapacity	46	5	25.9	28
Esteban	M	55	32	0	Incapacity	26	5	33.5	17
Monica	F	52	49	0	Incapacity	37	5	22.3	16
David	M	60	51	1	Incapacity	35	4	29.4	20
Edward	M	59	37	2	Incapacity	35	6	32.4	22
Abigail	F	76	62	2	Pensioner	31	5	33	14
Mia	F	59	27	4	Incapacity	29	5	31.8	22
Greta	F	49	30	1	Unemployment	43	5	28.8	23

BMI, Body Mass Index; CGI-S, Clinical Global Impressions–Severity; F, Female; MADRS, Montgomery-Åsberg Depression Rating Scale; M, Male; VO_2peak_, Peak Oxygen Uptake.

**Table 2:** Themes and subthemes from the analysis.

Theme	Subtheme
Intrapersonal factors	Clinical characteristics of the illness Lifestyle Medication Program perception
Interpersonal factors	External relationship Within-program relationships
Environmental factors	Location Timetable and duration FITT-PV principle Motivational elements

FITT-PV, frequency, intensity, time, type–progression, variety.

## Data Availability

The data presented in this study are available on request from the corresponding author due to ethical and privacy considerations, in accordance with the informed consent approved by the ethics committee.

## Abbreviations

The following abbreviations are used in this manuscript:

BMI	Body Mass Index
CGI-S	Clinical Global Impression
CRF	Cardiorespiratory fitness
FITT-PV	Frequency, intensity, time, type–progression, variety
LV-LIIT	Low-volume and low-intensity interval training
MADRS	Montgomery-Åsberg Depression Rating Scale
RMD	Resistant major depression
VO_2peak_	Peak oxygen uptake
VT1	First ventilatory threshold
VT2	Second ventilatory threshold
WHO	World Health Organization
